# MRI-based radiomics analysis improves preoperative diagnostic performance for the depth of stromal invasion in patients with early stage cervical cancer

**DOI:** 10.1186/s13244-022-01156-0

**Published:** 2022-01-29

**Authors:** Jing Ren, Yuan Li, Jun-Jun Yang, Jia Zhao, Yang Xiang, Chen Xia, Ying Cao, Bo Chen, Hui Guan, Ya-Fei Qi, Wen Tang, Kuan Chen, Yong-Lan He, Zheng-Yu Jin, Hua-Dan Xue

**Affiliations:** 1 Department of Radiology, Peking Union Medical College Hospital, Peking Union Medical College and Chinese Academy of Medical Sciences, Shuai Fu Yuan 1#, Dongcheng Dist., Beijing, 100730 People’s Republic of China; 2 Department of OB&GYN, Peking Union Medical College Hospital, Peking Union Medical College and Chinese Academy of Medical Sciences, Beijing, People’s Republic of China; 3 Beijing Infervision Technology Co., Ltd., Beijing, 100000 People’s Republic of China; 4 Department of Pathology, Peking Union Medical College Hospital, Peking Union Medical College and Chinese Academy of Medical Sciences, Beijing, People’s Republic of China; 5 Department of Radiotherapy, Peking Union Medical College Hospital, Peking Union Medical College and Chinese Academy of Medical Sciences, Beijing, People’s Republic of China

**Keywords:** Cervical cancer, Stromal invasion, Magnetic resonance imaging, Radiomics, Risk factor

## Abstract

**Background:**

The depth of cervical stromal invasion is one of the important prognostic factors affecting decision-making for early stage cervical cancer (CC). This study aimed to develop and validate a T2-weighted imaging (T2WI)-based radiomics model and explore independent risk factors (factors with statistical significance in both univariate and multivariate analyses) of middle or deep stromal invasion in early stage CC.

**Methods:**

Between March 2017 and March 2021, a total of 234 International Federation of Gynecology and Obstetrics IB1-IIA1 CC patients were enrolled and randomly divided into a training cohort (*n* = 188) and a validation cohort (*n* = 46). The radiomics features of each patient were extracted from preoperative sagittal T2WI, and key features were selected. After independent risk factors were identified, a combined model and nomogram incorporating radiomics signature and independent risk factors were developed. Diagnostic accuracy of radiologists was also evaluated.

**Results:**

The maximal tumor diameter (MTD) on magnetic resonance imaging was identified as an independent risk factor. In the validation cohort, the radiomics model, MTD, and combined model showed areas under the curve of 0.879, 0.844, and 0.886. The radiomics model and combined model showed the same sensitivity and specificity of 87.9% and 84.6%, which were better than radiologists (sensitivity, senior = 75.7%, junior = 63.6%; specificity, senior = 69.2%, junior = 53.8%) and MTD (sensitivity = 69.7%, specificity = 76.9%).

**Conclusion:**

MRI-based radiomics analysis outperformed radiologists for the preoperative diagnosis of middle or deep stromal invasion in early stage CC, and the probability can be individually evaluated by a nomogram.

## Key points

- Radiomics features are valuable in the preoperative diagnosis of stromal invasion depth.
- Maximum tumor diameter on MRI is a risk factor of stromal invasion.
- A nomogram incorporating radiomics signature and risk factors can facilitate clinical use.

## Introduction

Cervical cancer (CC) constitutes a heavy burden on women's health globally. It is the fourth most frequently occurring female malignancy and the fourth most common cause of cancer-related deaths. Approximately 604,127 new cases of CC are reported annually, with 341,831 deaths worldwide [1, 2]. The treatment for early stage (2018 International Federation of Gynecology and Obstetrics (FIGO) IB-IIA) CC includes surgery and primary chemoradiotherapy, and the determination of treatment strategies is largely dependent on tumor-related prognostic factors [3, 4], including the depth of cervical stromal invasion (DOI) [5, 6]. According to the Querleu-Morrow classification of radical hysterectomy, early stage CC patients without middle or deep 1/3 stromal invasion and a tumor size less than 2 cm can opt to undergo limited radical hysterectomy to avoid complications [7, 8]. However, most patients with middle or deep 1/3 stromal invasion are usually treated with radical hysterectomy and adjuvant radiotherapy, especially in the presence of other risk factors, such as special pathological types (adenocarcinoma, adenosquamous carcinoma, and neuroendocrine carcinoma, etc.), lymphovascular space invasion, or a large tumor size [3, 9]. In addition, concurrent radiochemotherapy is recommended over surgery for patients with middle or deep 1/3 stromal invasion and risk factors mentioned above, as it can achieve equal treatment efficacy with the combination of radical hysterectomy and adjuvant radiotherapy, and can avoid surgery-related adverse effects [5, 10–12]. Though the most accurate diagnosis of DOI is currently obtained through postoperative pathological analysis, novel preoperative approaches to accurately diagnose DOI is imperative to provide more reliable evidence for decision-making and to improve confidence in the management of patients with early stage CC.

Experience-related conventional imaging methods with limited accuracies, such as ultrasound, magnetic resonance imaging (MRI), and positron emission tomography-computed tomography (PET/CT), can be used to diagnose the DOI of early stage CC before treatment [13–15]. Transvaginal sonography has shown 63.6% accuracy, while MRI showed variable diagnostic sensitivities of 70% for radiologists with 7 years of experience in gynecologic cancer imaging, but this was only 50% for radiologists with 4 years of experience [13, 14]. PET/CT is not an ideal method for diagnosing DOI due to its high false-positivity rate of 40.8% [15]. Previous studies have found that some clinical characteristics, including tumor diameter on imaging, were associated with the DOI in patients with early stage CC, but their value in preoperative diagnosis of middle or deep 1/3 stromal invasion is yet to be investigated [16, 17].

Another preoperative method that may be applied for the diagnosis of DOI is radiomics. Radiomics analysis can convert medical images into high-dimensional, mineable data to evaluate tumor heterogeneity that cannot be identified by gross observation. The combination of radiomics-derived data and clinical data is a promising approach to enhance clinical management [18]. Since MRI is the best imaging modality to describe the extent of CC for treatment planning, several MRI-based radiomics analyses have been performed to predict prognostic factors (e.g., lymph node metastasis, parametrial invasion, and lymphovascular space invasion), treatment response, and patient survival [19–26]. Similar to clinical characteristics, limited radiomics studies aiming for preoperative diagnosis of middle or deep 1/3 stromal invasion in early stage CC have been conducted.

Therefore, in this study, we explored the value of MRI-based radiomics analysis and clinical characteristics in the preoperative diagnosis of middle or deep 1/3 stromal invasion. The study aimed to develop and validate diagnostic models to facilitate decision-making in the management of patients with early stage CC.

## Materials and methods

### Patients

Our institutional review board approved this retrospective study, so the requirement for informed consent was waived. This study was conducted and prepared by following the TRIPOD statement and CLAIM guideline for artificial intelligence [27, 28]. Patients with a clinical staging of FIGO IB1–IIA1 CC, as defined by the 2018 FIGO staging system, who underwent radical hysterectomy and pelvic lymph node dissection at our institution between March 2017 and March 2021 were enrolled in this study. The inclusion criteria were as follows: (1) pelvic MRI examination including sagittal T2-weighted imaging (T2WI) was performed within 14 days before surgery; (2) pathologic evaluation of the DOI was attainable; and (3) preoperative biopsy confirmed squamous cell carcinoma (SCC), adenocarcinoma (AC), or adenosquamous carcinoma (ASC). Patients were excluded for the following reasons: (1) patients who underwent treatment, such as neoadjuvant chemotherapy, radiotherapy, or cervical conization, prior to MRI examination or between MRI and surgery; (2) tumors were invisible on sagittal T2WI (considering the challenges to determine the ROIs of invisible tumors); and (3) insufficient image quality to extract radiomics features. A total of 234 patients were included in the study. A flowchart of this study is presented in Fig©1. Eligible patient data were randomly divided into a training cohort (188 patients, mean age 44.63 ± 9.61 years) and a validation cohort (46 patients, mean age 46.56 ± 10.36 years), at a ratio of 8:2. The clinical characteristics of all enrolled patients, including age, 2018 FIGO stage, menopausal status, preoperative biopsy histological type, and maximal tumor diameter (MTD) on MR images that the lesions appeared largest, were obtained from medical records. Clinical and imaging records of the patients before 2018 were reviewed by a gynecological oncologist with 10 years of experience and were restaged according to the 2018 FIGO staging criteria [29].

**Fig. 1:**
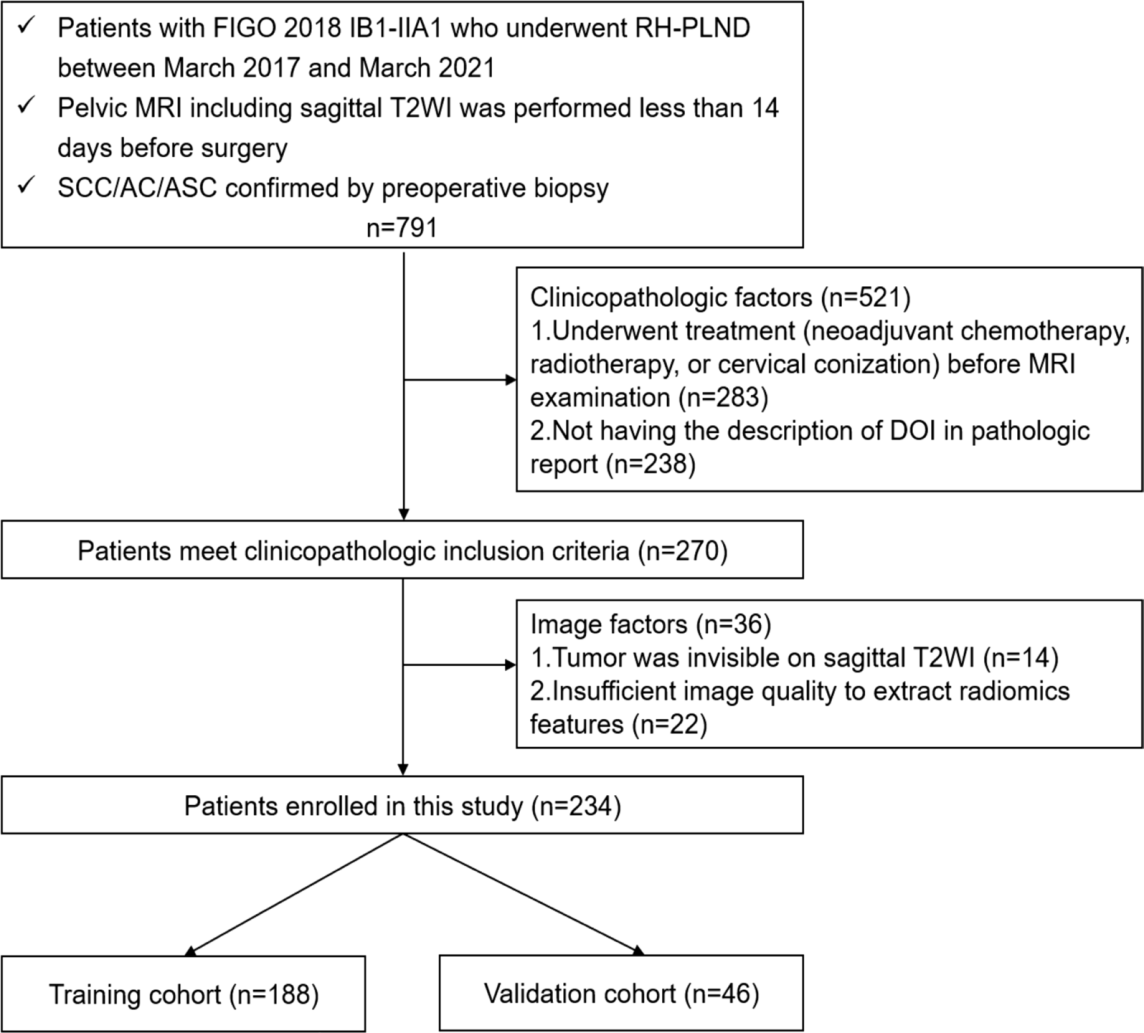
Flowchart of the study

Postoperative pathological examination results were the gold standard in this study. DOI was measured as a percentage of the tumor depth to the cervical radius in millimeters (mm) and was recorded in the pathology report as “tumor depth/cervical radius (mm)” [6]. Superficial 1/3 stromal invasion was defined as “superficial stromal invasion” and middle or deep 1/3 stromal invasion as “middle or deep stromal invasion” [9].

### MRI acquisition

Preoperative pelvic MRI examinations were performed using a Signa Excite 1.5 T scanner for 140 patients, a Discovery MR 750 W 3 T scanner for 64 patients, and a Discovery MR 750 3 T scanner for 30 patients (GE Medical Systems, Milwaukee, WI, USA). Pelvic MRI was performed with supine body array coils. A total of 216 patients received intravenous contrast material, and the enhanced images were acquired. T2WI was the mainstay for the detection of tumor size and extent of local disease. Sagittal T2W images were selected to extract radiomics features considering their greater consistency across multiple centers compared to other T2W planes. The detailed sagittal T2W MRI acquisition parameters of each device were as follows: Signa Excite 1.5 T (TR/TE, 3400/88 ms; FOV, 270 × 270 mm^2^; matrix, 288 × 192; slice thickness/gap, 5.5/1 mm), Discovery MR 750 W 3 T (TR/TE, 4273/79 ms; FOV, 280 × 280 mm^2^; matrix, 288 × 192; slice thickness/gap, 4.5/1 mm), and Discovery MR 750 3 T (TR/TE, 3607/111 ms; FOV, 220 × 220 mm^2^; matrix, 288 × 192; slice thickness/gap, 5/1 mm). Sagittal T2W digital imaging and communications in medicine (DICOM) images of all patients were retrieved from the picture archiving and communication system for image feature extraction.

### Image segmentation

The InferScholar Center software (Infervision Medical Technology Co., Ltd., version 3.2) was used for three-dimensional manual segmentation [30]. Firstly, the initial DICOM image dataset of each patient was anonymized and then uploaded to the software. Then a radiologist with 10 years of experience in gynecological MRI interpretation delineated the region of interest (ROI) along the tumor contour on each sagittal T2WI slice. In the process, the radiologist also referred to other sequences of images (T1W and diffusion-weighted imaging) to ensure the accuracy of image segmentation. Each segmentation was subsequently validated by a senior radiologist with 17 years of experience in gynecological MRI interpretation. Any disagreement over segmentation was resolved by a consultation to reach a consensus. Both radiologists were aware of the CC diagnosis, but they were blinded to the clinical and histopathological data.

### Radiomics feature extraction

After manual segmentation, the original DICOM images and segmentation results were normalized according to pixel spacing and slice thickness to reduce the influence of various acquisition parameters of different MR image systems on the stability of radiomics features [31]. Subsequently, radiomics features, including first-order features, shape-based features, gray level co-occurrence matrix (GLCM) features, gray level dependence matrix (GLDM) features, gray level run length matrix (GLRLM) features, gray level size zone matrix (GLSZM) features, and neighboring gray-tone difference matrix (NGTDM) features were extracted from each ROI. Shape-based features were extracted from the original images, and the other six sets of features were extracted from both the original and processed images. Then, each feature was standardized using z-score normalization to obtain a standard image intensity normal distribution, thereby facilitating good feature robustness [32]. Pycharm (version 2019.1.3; https://www.jetbrains.com/) was used for the normalization of the original images and ROI and for extraction of radiomics features.

### Feature selection and radiomics model building

Since not all extracted radiomics features correlated with middle or deep stromal invasion, a three-step feature selection was performed to verify important features with high predictive powers. First, a significance test for each feature was conducted, and features with statistical significance (*p* < 0.05) were retained. Second, all retained features were matched pairwise, and if the Pearson correlation coefficient between two features was > 0.85, then the feature with the higher *p* value in the significance test was eliminated, and the remaining features were processed as follows. Finally, a fivefold cross-validation-based least absolute shrinkage and selection operator (LASSO) was applied to select features with nonzero coefficients from the remaining features. LASSO regularization involves parameter λ to control the number of selected features. In this study, the optimal λ was selected as the lowest binomial deviance in the training cohort data, consequently retaining a relatively small number of features to fit further models. After radiomics feature selection, a radiomics model based on logistic regression was built using the training cohort data to predict middle or deep stromal invasion. Radiomics feature selection and logistic regression model building used Rstudio (V.3.5.0; https://www.r-project.org/).

### Independent risk factor identification and combined model building

Independent risk factors for predicting middle or deep stromal invasion were identified by univariate and multivariate analysis of the patient data for five clinical characteristics (age, 2018 FIGO stage, menopausal status, preoperative biopsy histological type, and MTD on MRI). A combined model incorporating the independent risk factors and selected radiomics features was built using training cohort data.

### Development of the nomogram

To clinically apply the diagnostic models, a multivariable logistic regression analysis was applied to build a nomogram based on the training cohort data that visually represented the combined model. The nomogram serves as an individual tool that integrates the radiomics signature with independent risk factors to predict the probability of middle or deep stromal invasion in early stage CC. The radiomics signature in the nomogram was the linear sum of the selected radiomics features and their corresponding coefficients.

### Subjective evaluation by radiologists

To compare the diagnostic performance of the radiomics models and radiologists, two radiologists with 5 and 10 years of experience in gynecological MRI interpretation independently determined the presence of middle or deep stromal invasion for each patient in validation cohort (*n* = 46). They were blind to the patient information but were aware of the CC diagnosis, and they made the decision by browsing through all the MR sequences.

### Statistical analysis

Statistical analysis was conducted using R version 3.5.0 (https://www.r-project.org/) and SPSS version 21.0.0.0 (https://www.ibm.com). The independent sample t test was used to compare the differences in continuous variables (age and MTD on MRI) between the superficial stromal invasion and middle or deep stromal invasion groups, as well as the training and validation cohorts. The chi-square test was used to evaluate the significance of categorical variables, including 2018 FIGO stage, menopausal status, and preoperative biopsy histological type between the superficial and middle or deep stromal invasion groups. The difference in the prevalence of middle or deep stromal invasion between the training and validation cohorts was also compared using the chi-square test. Fisher’s exact test was used for variables with a frequency of less than five. Independent risk factors for middle or deep stromal invasion were identified using multivariate logistic regression analysis with the forward Wald method by inputting significant variables found by univariate analysis. The area under the curve (AUC) of the receiver operating characteristic (ROC) curve was calculated to assess the diagnostic performance of the radiomics model, independent risk factors, and the combined model in the validation cohort. Their sensitivity, specificity, positive predictive value (PPV), and negative predictive value (NPV) were calculated to further evaluate their performance, and 95% confidence intervals (CIs) were estimated using 1000-replicate bootstrapping. Interobserver agreement of the two radiologists was evaluated by Cohen’s κ coefficient test (< 0.20, poor agreement; 0.21–0.40, fair agreement; 0.41–0.60, moderate agreement; 0.61–0.80, substantial agreement; > 0.80, almost perfect agreement). Statistical significance was set at *p* < 0.05.

## Results

### Clinical characteristics

Among the 234 enrolled patients, 70 (29.9%) had superficial stromal invasion and 164 (70.1%) had middle or deep stromal invasion. There was no significant difference in the middle or deep stromal invasion prevalence between the training and validation cohorts (*p* = 0.859). Premenopausal cases accounted for 70.5% of all patients. The percentages of patients with IB1, IB2, IB3, and IIA1 CC were 41.4%, 50.9%, 3.0%, and 4.7%, respectively. The majority of patients (69.2%) had preoperatively proven SCC. MTD on MRI was 23.10 ± 9.28 mm (range 5.8–63.0 mm). There were no significant differences in clinical characteristics between the training and validation cohorts (*p* > 0.05) (Table 1).

**Table 1:** Characteristics of patients in training and validation cohorts

	Training cohort (*n* = 188)			Validation cohort (*n* = 46)			P*
	Superficial stromal invasion	Middle or deep stromal invasion	*p* value	Superficial stromal invasion	Middle or deep stromal invasion	*p* value	
	(*n* = 57)	(*n* = 131)		(*n* = 13)	(*n* = 33)		
Age, mean ± SD, years	43.81 ± 9.88	44.98 ± 9.50	0.441	46.00 ± 9.49	46.79 ± 10.82	0.819	0.229
Menstrual status (N. %)			0.327			1.000	0.279
Premenopausal	44	92		8	21		
Postmenopausal	13	39		5	12		
FIGO stage (N. %)			< 0.001			0.028	0.985
IB1	41	36		10	10		
IB2	15	81		3	20		
IB3	0	6		0	1		
IIA1	1	8		0	2		
Biopsy histological type (N. %)			0.553			0.862	0.729
SCC	37	92		9	24		
AC	19	34		4	7		
ASC	1	5		0	2		
MTD on MRI, mean ± SD, mm	17.63 ± 7.03	25.82 ± 9.13	< 0.001	14.65 ± 6.16	25.06 ± 8.39	< 0.001	0.469

*P* is derived from the univariable association analysis of each clinical variable between superficial stromal invasion patients and middle or deep stromal invasion patients in the training and validation cohort, respectively. P^*^ represents the difference of each clinical variable between the training and validation cohorts

SD, standard deviation; FIGO, Federation International of Gynecology and Obstetrics; SCC, squamous cell carcinoma; AC, adenocarcinoma; ASC, adenosquamous carcinoma; MTD, maximal tumor diameter

### Radiomics feature selection and radiomics model building

**Fig. 2:**
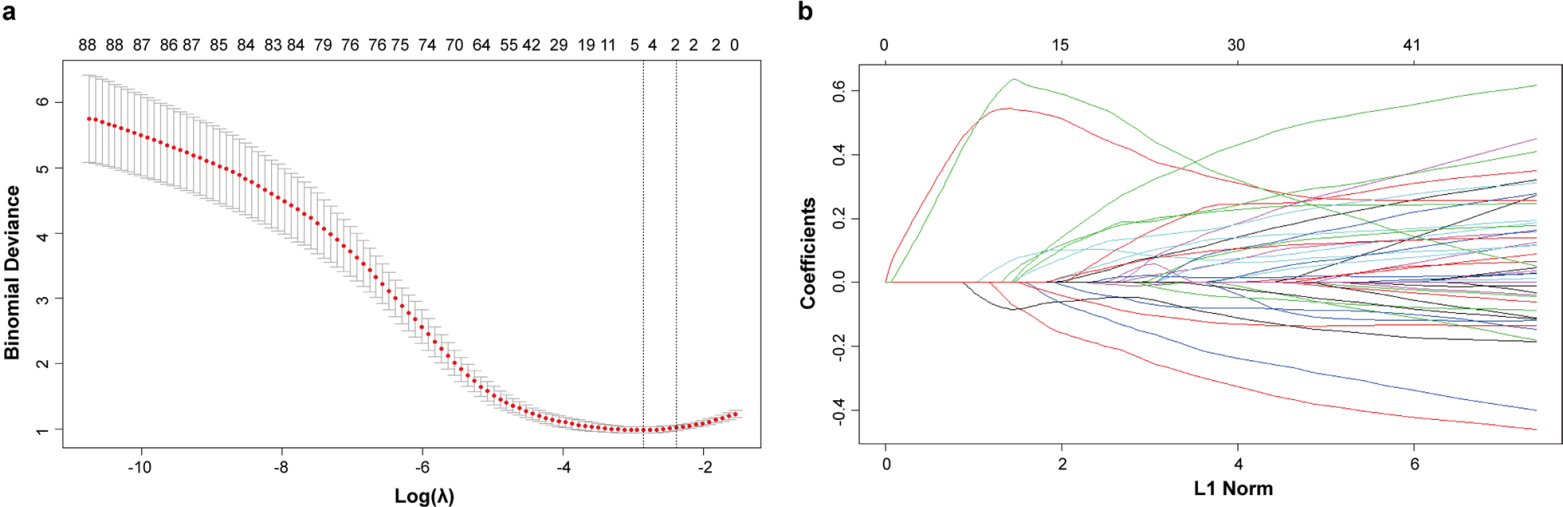
Radiomics feature selection using the least absolute shrinkage and selection operator (LASSO) regression method. **a** The optimal *λ* was selected as the lowest binomial in the LASSO model using fivefold cross-validation. **b** LASSO coefficient profiles of the features show vertical lines that are drawn at the value selected using fivefold cross-validation, and the optimal *λ* results in 5 nonzero coefficients

In the training cohort, 1,454 radiomics features were extracted from each segmented tumor volume on sagittal T2 images of each patient after original image and ROI normalization. To reduce the risk of over-fitting, the three-step feature selection process based on the training cohort data led to the exclusion of non-relevant and redundant features. The first step retained 944 radiomics features with statistical significance (*p* < 0.05) between superficial stromal invasion group and middle or deep stromal invasion group. Then redundant features were discarded and 130 representative features were proceeded to the following process. Finally, the optimal λ was selected and consequently led to the selection of five significant radiomics features for the prediction of middle or deep stromal invasion (Fig. 2). These radiomics features were glcm_Idmn_wavelet.LHL, glcm_Imc2_wavelet.LLH, gldm_SmallDependenceLowGrayLevelEmphasis_wavelet.HHH, glrlm_LongRunEmphasis_wavelet.HHH, and shape_LeastAxisLength_original. A radiomics model was built using the five selected radiomics features. The model showed a good diagnostic performance for middle or deep stromal invasion with an AUC of 0.879 (0.775–0.983), a sensitivity of 87.9%, and a specificity of 84.6% in the validation cohort. The model had a NPV of 73.3% and a PPV of 93.5% in the validation cohort.

### Independent risk factor identification and combined model building

**Fig. 3:**
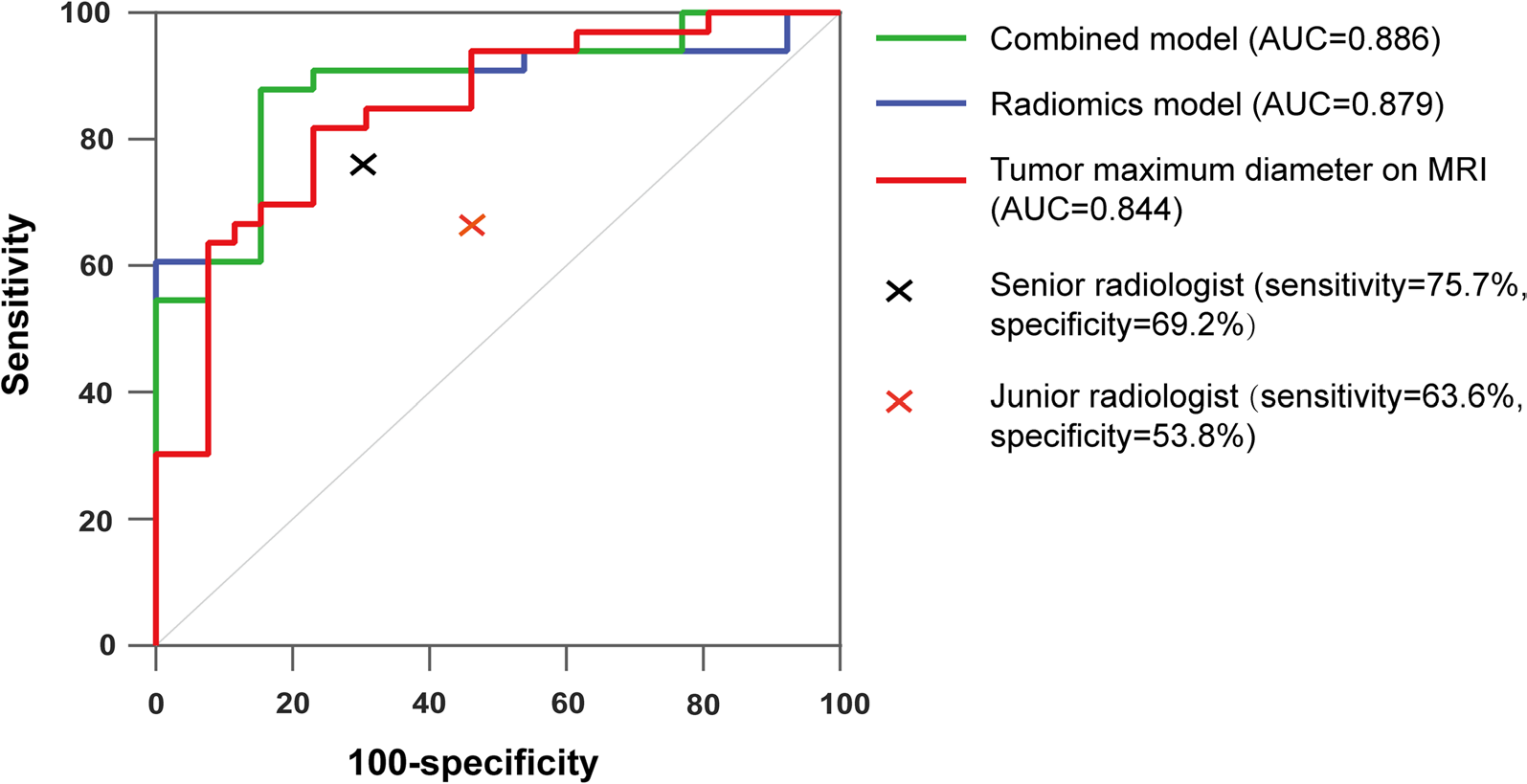
ROC curves of combined model, radiomics model, and tumor maximum diameter on MRI for predicting middle or deep stroma invasion in the validation cohort. The senior radiologist’s performance is indicated by the black cross and the junior radiologist’s performance is indicated by the red cross

Univariate analysis showed that 2018 FIGO stage and MTD on MRI were significant risk factors for middle or deep stromal invasion. Multivariate logistic regression analysis showed that only MTD on MRI was an independent risk factor for middle or deep stromal invasion (Table 2). When the cut-off value of MTD on MRI was 22.1 mm, the AUC, sensitivity, and specificity were 0.844 (0.719–0.969), 69.7%, and 76.9%, respectively, to predict middle or deep stromal invasion. A combined model was also built using MTD on MRI and five selected radiomics features, which yielded an AUC, sensitivity, and specificity of 0.886 (0.784–0.988), 87.9%, and 84.6%, respectively. The results are shown in Table 3, and the ROC curves are shown in Fig. 3.

**Table 2:** Univariate and multivariate logistic regression analysis for independent risk factors of middle or deep stromal invasion

Variables	Univariate analysis			Multivariate analysis		
	Odds ratio	95% CI	*p* value	Odds ratio	95% CI	*p* value
Age	1.012	0.983–1.042	0.416	–	–	–
Menopause status	1.304	0.695–2.448	0.409	–	–	–
FIGO stage	5.085	2.826–9.149	< 0.001	1.380	0.655–2.910	0.397
Biopsy histological type	0.929	0.557–1.550	0.778	–	–	–
MTD on MRI	1.156	1.103–1.213	< 0.001	1.131	1.058–1.210	< 0.001

FIGO, Federation International of Gynecology and Obstetrics; CI, confidence interval; MTD, maximal tumor diameter

**Table 3:** Diagnostic performance of radiomics model, combined model, MTD on MRI, and radiologists in validation cohort

	AUC	SEN	SPE	PPV	NPV
Radiomics Model	0.879 (0.775–0.983)	0.879 (0.727–0.952)	0.846 (0.578–0.973)	0.935 (0.772–0.989)	0.733 (0.448–0.911)
Combined Model	0.886 (0.784–0.988)	0.879 (0.727–0.952)	0.846 (0.578–0.973)	0.935 (0.772–0.989)	0.733 (0.448–0.911)
MTD on MRI	0.844 (0.719–0.969)	0.697 (0.527–0.826)	0.769 (0.497–0.918)	0.885 (0.687–0.970)	0.500 (0.279–0.721)
Senior radiologist	–	75.7%	69.2%	86.2%	52.9%
Junior radiologist	–	63.6%	53.8%	77.8%	36.8%

AUC, Area Under the Curve; SEN, sensitivity; SPE, specificity; PPV, positive predictive value; NPV, negative predictive value; MTD, maximal tumor diameter

### Development of the nomogram

**Fig. 4:**
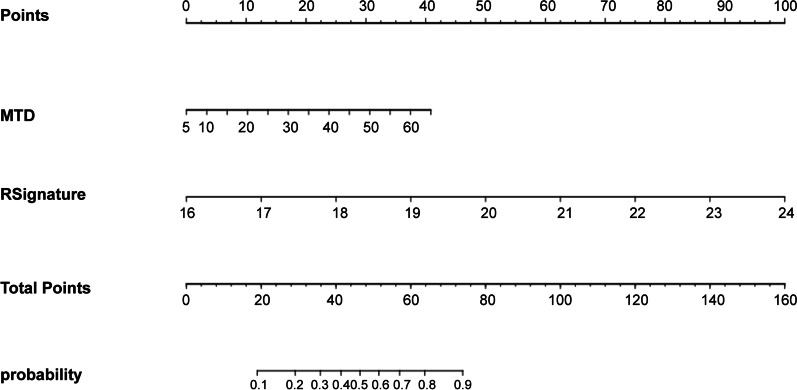
Nomogram for individual prediction of the probability of middle or deep stroma invasion in early stage CC. The nomogram was was a visual representation of the combined model in training cohort, which integrated radiomics signature and independent risk factor. The radiomics signature in the nomogram was the linear sum of the selected 5 radiomics features and their corresponding coefficients. (Rsignature: radiomics signature; MTD: maximal tumor diameter on MRI)

**Fig. 5:**
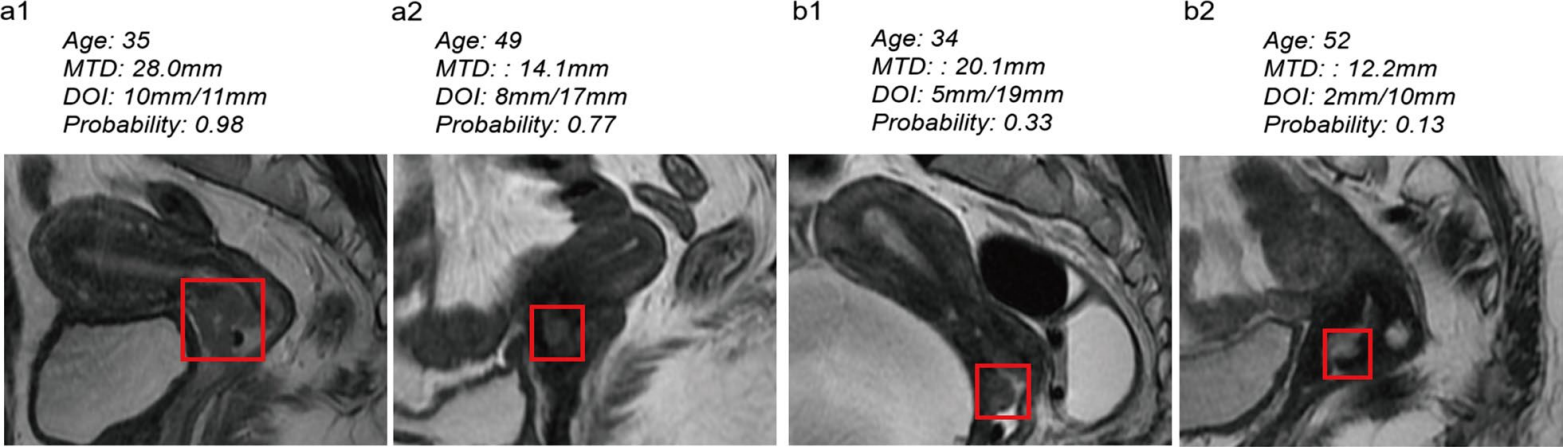
Representative images of middle or deep cervical stroma invasion (**a**) and superficial cervical stroma invasion (**b**). The lesions in the frames on sagittal T2WI are cervical tumors. **a1** a 35-year-old, 2018 FIGO IB2, SCC patient with MTD on MRI of 28.0 mm. The probability of the middle or deep stroma invasion predicted by the nomogram was 98%. **a2** a 49-year-old, 2018 FIGO IB1, SCC patient with MTD on MRI of 14.1 mm. The probability of the middle or deep stroma invasion predicted by the nomogram was 77%. **b1** a 34-year-old, 2018 FIGO IB2, SCC patient with MTD on MRI of 20.1 mm. The probability of the middle or deep stroma invasion predicted by the nomogram was 33%. **b2** a 52-year-old, 2018 FIGO IB1, AC patient with MTD on MRI of 12.2 mm. The probability of the middle or deep stroma invasion predicted by the nomogram was 13%. (MTD: maximal tumor diameter; FIGO: Federation International of Gynecology and Obstetrics; SCC: squamous cell carcinoma; AC: adenocarcinoma)

The radiomics signature and MTD on MRI were used to develop a nomogram. The nomogram is shown in Fig. 4. Representative images of middle or deep cervical stromal invasion and superficial cervical stromal invasion are shown in Fig. 5.

### Subjective evaluation by radiologists

The interobserver agreement of the two radiologists was fair (*κ* = 0.362). The diagnostic accuracy was 73.9% for the senior and 60.9% for the junior. Of the 33 patients with the presence of middle or deep stromal invasion, the junior radiologist identified 21 patients and the senior radiologist identified 25 patients. The sensitivity and PPV of the two radiologists were 63.6% and 77.8%, and 75.7% and 86.2%, respectively. The senior radiologist identified more patients without middle or deep stromal invasion (9/13) than the junior radiologist (7/13), with specificities and NPVs of 69.2% and 52.9% for the senior radiologist, and 53.8% and 36.8% for the junior radiologist (Table 3).

## Discussion

A radiomics model based on T2W images is valuable in the preoperative diagnosis of middle or deep stromal invasion of early stage CC. MTD on MRI was shown to be an independent risk factor of middle or deep stromal invasion. A nomogram was constructed to facilitates the clinical application for the individual prediction of the middle or deep stromal invasion probability in patients with early stage CC.

Accurate preoperative diagnosis of middle or deep stromal invasion contributes to optimal treatment decision-making, and facilitate doctor-patient communication on treatment strategy selection. For early stage CC patients, surgery is recommended only when the patient is deemed to have no indication for adjuvant chemoradiotherapy [12]. Among 234 patients included in this study, middle or deep stromal invasion was present in 164 (70.1%) patients, indicating that a significant portion of early stage CC patients are at risk for adjuvant chemoradiotherapy after surgery, especially if other risk factors are present. Based on the tradition MR images, the variability of DOI diagnosis was substantially large among radiologists with different seniority. Furthermore, the sensitivity and specificity of the radiologists were lower than that of the radiomics model based on sagittal T2WI. The results suggest that the radiomics model has the potential to assist in clinical settings by reducing the rate of misdiagnoses in middle or deep stromal invasion and improving confidence in decision-making in oncologic management. Additionally, the better negative predictive value and positive predictive value of the radiomics model verified its potential to assist radiologists and oncologists in doctor-patient communication, which means that if the patient is diagnosed as middle or deep stromal invasion by the radiomics model, primary chemoradiotherapy rather than surgery may be proposed more confidently by the clinicians.

To identify the independent risk factors of middle or deep stromal invasion, univariate and multivariate analyses on clinical characteristics were performed. MTD on MRI was the only independent risk factor for middle or deep stromal invasion in early stage CC. The results were consistent with a previous study, where the authors found that when the cut-off value was 20.5 mm, two-dimensional MTD on ultrasound showed good performance in predicting deep stromal invasion with an AUC of 0.83, a sensitivity of 90.5%, and a specificity of 61.1% [17]. However, the combined model incorporating a radiomics signature and MTD on MRI only achieved a slight improvement in AUC and the same sensitivity and specificity compared to that of our proposed radiomics model. This suggests that tumor size correlates with DOI but has limited predictive value.

The FIGO staging system is considered the most powerful tool in treatment planning and counseling patients regarding the prognosis of CC, and the latest 2018 FIGO allowed imaging findings to stage the disease for the first time [33]. In previous study, radiomics model for survical prediction in CC patients showed better performance when FIGO stage was added [34]. In the current study, the 2018 FIGO stage was not incorporated in the combined model because it showed significant differences between the superficial and middle or deep stromal invasion groups in univariate analysis, but not in multivariate analysis. A possible reason is that the 2018 FIGO staging of early CC includes some information on tumor diameter, but the information is limited as tumor size is only divided into 2 cm or 4 cm.

Radiomics features were extracted from the mainstay T2WI sequence, since it provides indications of disease extent and the relationship of the tumor with surrounding tissues in CC [35]. Tumor segmentation is a critical step in radiomics analysis flow. Multiple-segmentation by multiple clinicians is a method to provided reproducible and reliable radiomic features. As reported in previous studies, 95.2% of radiomics features extracted from T2WI showed high reproducibility (intraclass correlation coefficient ≥ 0.75) in different CC segmentations contoured by three observers [36]. Considering that, interobserver segmentation variability was not provided in the present study, but each segmented volumes were carefully validated by a senior radiologist to ensure that the radiomic features are reliable. In addition, a real-world patient cohort was included to reduce selection bias, but the large majority of patients with CC in the present study were excluded considering the potential impact of the long interval and preoperative treatments on the accuracy of pathological results. Three different MRI devices were implemented in the real-world patient cohort, which may had a potential impact on the stability of radiomics features. Thus, the normalized DICOM image and segmentation results allowed each feature to have the same mean of 0 and a standard deviation of 1, contributing to the normal distribution [31, 37]. The standardization process was considered a useful method to facilitate good feature robustness in CC, which was maintained for 94.4–100% of T2W radiomics features [32]. Moreover, the use of high-dimensional features contributed to the good performance of the model. Four of the five selected features obtained after wavelet transformation are high-dimensional features. These features were difficult to decipher through simple observation, but they provide considerably richer information about the intensity, shape, size, and volume of cervical tumors.

There are several limitations to this study. First, all patients were recruited from a single center with a relatively small sample size. Although imaging data with different scanning parameters were included, the exclusion of large majority of CC patients in our institution may increase the risk of selection bias. The diagnostic performance of the radiomics model should be validated with a larger multicenter data in the future. Second, the radiomics features were only derived from sagittal T2W images. Previous studies showed the value of diffusion-weighted images and contrasted-enhanced images in predicting prognostic factors such as lymph node metastasis and lymphovascular space invasion in CC patients [20, 21]. Finally, the ROI segmentation was only conducted by one radiologist in this study. The extracted radiomics features robustness to segmentation variabilities was unknown, and the overfitting risk caused by irreproducible features existed. Therefore, radiomics studies on multiple segmentations are necessary.

In conclusion, radiomics model based on T2WI outperformed radiologists in the preoperative diagnosis of middle or deep stromal invasion in patients with early stage CC. MTD on MRI is an independent risk factor for middle or deep stromal invasion but has limited sensitivity and specificity. In addition, the nomogram incorporating a radiomics signature and MTD on MRI can evaluate the probability of middle or deep stromal invasion for early stage CC.

## Data Availability

The datasets analyzed during the current study are available from the corresponding author on reasonable request.

## Abbreviations

AC: Adenocarcinoma
ASC: Adenosquamous carcinoma
AUC: Area under the curve
CC: Cervical cancer
CIs: Confidence intervals
DICOM: Digital imaging and communications in medicine
DOI: Depth of stromal invasion
FIGO: International federation of gynecology and obstetrics
LASSO: Least absolute shrinkage and selection operator
MTD: Maximal tumor diameter
ROC: Receiver-operating characteristic
ROI: Region of interest
SCC: Squamous cell carcinoma

## References

[1] Sung H, Ferlay J, Siegel RL (2021). Global cancer statistics 2020: GLOBOCAN estimates of incidence and mortality worldwide for 36 cancers in 185 countries. CA Cancer J Clin.

[2] Shrestha AD, Neupane D, Vedsted P, Kallestrup P (2018). Cervical cancer prevalence, incidence and mortality in low and middle income countries: a systematic review. Asian Pac J Cancer Prev.

[3] Cibula D, Pötter R, Planchamp F (2018). The European Society of Gynaecological Oncology/European Society for Radiotherapy and Oncology/European Society of Pathology guidelines for the management of patients with cervical cancer. Radiother Oncol.

[4] Bhatla N, Denny L (2018). FIGO cancer report 2018. Int J Gynaecol Obstet.

[5] Cao L, Wen H, Feng Z, Han X, Zhu J, Wu X (2021). Role of adjuvant therapy after radical hysterectomy in intermediate-risk, early-stage cervical cancer. Int J Gynecol Cancer.

[6] Allam M, Feely C, Millan D, Nevin J, Davis J, Siddiqui N (2004). Depth of cervical stromal invasion as a prognostic factor after radical surgery for early stage cervical cancer. Gynecol Oncol.

[7] Querleu D, Morrow CP (2008). Classification of radical hysterectomy. Lancet Oncol.

[8] Querleu D, Cibula D, Abu-Rustum NR (2017). 2017 Update on the Querleu-Morrow classification of radical hysterectomy. Ann Surg Oncol.

[9] National Comprehensive Cancer Network. NCCN Clinical Practice Guidelines in Oncology. Cervical Cancer (Version 1.2020). [cited 2020 Jan 14]. Available from: https://www.nccn.org/professionals/physician_gls/pdf/cervical.pdf

[10] Baalbergen A, Veenstra Y, Stalpers LL, Ansink AC (2010). Primary surgery versus primary radiation therapy with or without chemotherapy for early adenocarcinoma of the uterine cervix. Cochrane Database Syst Rev.

[11] Landoni F, Colombo A, Milani R, Placa F, Zanagnolo V, Mangioni C (2017). Randomized study between radical surgery and radiotherapy for the treatment of stage IB-IIA cervical cancer: 20-year update. J Gynecol Oncol.

[12] Marth C, Landoni F, Mahner S, McCormack M, Gonzalez-Martin A, Colombo N (2017). Cervical cancer: ESMO clinical practice guidelines for diagnosis, treatment and follow-up. Ann Oncol.

[13] Moloney F, Ryan D, Twomey M, Hewitt M, Barry J (2016). Comparison of MRI and high-resolution transvaginal sonography for the local staging of cervical cancer. J Clin Ultrasound.

[14] Lakhman Y, Akin O, Park KJ (2013). Stage IB1 cervical cancer: role of preoperative MR imaging in selection of patients for fertility-sparing radical trachelectomy. Radiology.

[15] Yang Z, Xu W, Ma Y, Liu K, Li Y, Wang D (2016). (18)F-FDG PET/CT can correct the clinical stages and predict pathological parameters before operation in cervical cancer. Eur J Radiol.

[16] Liu S, Xia L, Yang Z (2020). The feasibility of (18)F-FDG PET/CT for predicting pathologic risk status in early-stage uterine cervical squamous cancer. Cancer Imaging.

[17] Pálsdóttir K, Fischerova D, Franchi D, Testa A, Di Legge A, Epstein E (2015). Preoperative prediction of lymph node metastasis and deep stromal invasion in women with invasive cervical cancer: prospective multicenter study using 2D and 3D ultrasound. Ultrasound Obstet Gynecol.

[18] Lambin P, Rios-Velazquez E, Leijenaar R (2012). Radiomics: extracting more information from medical images using advanced feature analysis. Eur J Cancer.

[19] Wu Q, Wang S, Chen X (2019). Radiomics analysis of magnetic resonance imaging improves diagnostic performance of lymph node metastasis in patients with cervical cancer. Radiother Oncol.

[20] Xiao M, Ma F, Li Y (2020). Multiparametric MRI-based radiomics nomogram for predicting lymph node metastasis in early-stage cervical cancer. J Magn Reson Imaging.

[21] Li Z, Li H, Wang S (2019). MR-based radiomics nomogram of cervical cancer in prediction of the lymph-vascular space invasion preoperatively. J Magn Reson Imaging.

[22] Wu Q, Shi D, Dou S (2019). Radiomics analysis of multiparametric MRI evaluates the pathological features of cervical squamous cell carcinoma. J Magn Reson Imaging.

[23] Wang T, Gao T, Guo H (2020). Preoperative prediction of parametrial invasion in early-stage cervical cancer with MRI-based radiomics nomogram. Eur Radiol.

[24] Gui B, Autorino R, Miccò M (2021). Pretreatment MRI radiomics based response prediction model in locally advanced cervical cancer. Diagnostics (Basel).

[25] Fang M, Kan Y, Dong D (2020). Multi-habitat based radiomics for the prediction of treatment response to concurrent chemotherapy and radiation therapy in locally advanced cervical cancer. Front Oncol.

[26] Wormald BW, Doran SJ, Ind TE, D’Arcy J, Petts J, deSouza NM (2020). Radiomic features of cervical cancer on T2-and diffusion-weighted MRI: prognostic value in low-volume tumors suitable for trachelectomy. Gynecol Oncol.

[27] Collins GS, Reitsma JB, Altman DG, Moons KG (2015). Transparent reporting of a multivariable prediction model for individual prognosis or diagnosis (TRIPOD): the TRIPOD statement. BMJ.

[28] Mongan J, Moy L, Kahn CE (2020). Checklist for artificial intelligence in medical imaging (CLAIM): a guide for authors and reviewers. Radiol Artif Intell.

[29] Bhatla N, Berek JS, Cuello Fredes M (2019). Revised FIGO staging for carcinoma of the cervix uteri. Int J Gynaecol Obstet.

[30] Wang M, Xia C, Huang L (2020). Deep learning-based triage and analysis of lesion burden for COVID-19: a retrospective study with external validation. Lancet Digit Health.

[31] Wei W, Liu Z, Rong Y (2019). A computed tomography-based radiomic prognostic marker of advanced high-grade serous ovarian cancer recurrence: a multicenter study. Front Oncol.

[32] Park SH, Lim H, Bae BK (2021). Robustness of magnetic resonance radiomic features to pixel size resampling and interpolation in patients with cervical cancer. Cancer Imaging.

[33] Bhatla N, Aoki D, Sharma DN, Sankaranarayanan R (2018). Cancer of the cervix uteri. Int J Gynaecol Obstet.

[34] Li H, Zhu M, Jian L (2021). Radiomic score as a potential imaging biomarker for predicting survival in patients with cervical cancer. Front Oncol.

[35] Manganaro L, Lakhman Y, Bharwani N (2021). Staging, recurrence and follow-up of uterine cervical cancer using MRI: updated guidelines of the European Society of Urogenital Radiology after revised FIGO staging 2018. Eur Radiol.

[36] Fiset S, Welch ML, Weiss J (2019). Repeatability and reproducibility of MRI-based radiomic features in cervical cancer. Radiother Oncol.

[37] Rai R, Holloway LC, Brink C (2020). Multicenter evaluation of MRI-based radiomic features: a phantom study. Med Phys.

